# Technical Note: Tibial Spine Avulsion Treatment with Arthroscopic Reduction and Internal Fixation with Kirschner Wires in Skeletally Immature Patients

**DOI:** 10.3390/healthcare11172404

**Published:** 2023-08-27

**Authors:** Vittorio Calvisi, Emilio Romanini, Donato Staniscia, Giovanni Di Brigida, Michele Venosa

**Affiliations:** 1 Department of Life, Health and Environmental Sciences, University of L’Aquila, Via Vetoio Coppito 2, 67100 L’Aquila, Italy; vittorio.calvisi@univaq.it (V.C.);; 2 UOSD, Department of Mini-Invasive and Computer-Assisting Orthopedic Surgery, San Salvatore Hospital, Via L. Natali 1, 67100 L’Aquila, Italy; 3 RomaPro, Polo Sanitario San Feliciano, Via Mattia Battistini, 44, 00167 Rome, Italy; 4 GLOBE, Italian Working Group on Evidence-Based Orthopedics, Via Nicola Martelli, 3, 00197 Rome, Italy

**Keywords:** tibial spine fracture, tibial eminence fracture, arthroscopy, anterior cruciate ligament, osteosynthesis

## Abstract

Introduction: Tibial spine avulsion injury, tibial eminence injury, tibial spine fracture, and anterior cruciate ligament (ACL) avulsion are multiple terms that express the same pathological condition. It can be encountered both in the pediatric and adult population. A wide array of surgical techniques have been proposed to manage displaced tibial spine avulsions. Anyway, insufficient evidence is currently available to prefer one fixation technique over another, and a gold-standard arthroscopy-based technique is still missing. In this article, we describe a mini-invasive, safe and user-friendly technique for arthroscopic reduction and internal fixation of displaced tibial eminence fractures. Materials and methods: Standard and patient-specific accessory arthroscopic portals allow for full access to knee visualization and management of concomitant intraarticular lesions. After performing the debridement of the inflammatory tissue and the release of eventual interposed tissues in the fracture site, the tibial eminence avulsion can be reduced by using a less-invasive bone impactor. With the knee flexed to 90°, the fracture fragments are then synthesized (under fluoroscopic control) with three thin Kirschner wires inserted in a proximal–distal direction in a cross-shaped geometry. Results: This technique allows a fast surgical and hospitalization time, a punctiform arthrotomy, proximal tibial physis preservation, and an early rehabilitation program. Conclusions: This novel technique seems attractive and very promising since it is respectful of the epiphyseal growth plates and is thus suitable for children and adolescents.

## 1. Introduction

Tibial spine avulsions (TSAs), once considered a rare event in the pediatric population, are actually relatively frequent in adolescents, with an annual incidence of 3 per 100,000 [1,2,3] as a consequence of a sports injury (e.g., skiing, cycling) but they can be encountered even in adults as a consequence of motor vehicle accidents [4,5,6]. Tibial spine avulsion injury, tibial eminence injury, tibial spine fracture, and anterior cruciate ligament (ACL) avulsion are multiple terms that express the same pathological condition. The rate of force applied to the knee with the tibia in internal rotation and the knee protected from the tension of the flexor muscles is assumed to be responsible for the fracture at the tibial eminence [3,7,8].

Anamnestic evaluation is very similar to an ACL mid-substance rupture, with noisy crunch knee pain, immediate joint effusion, and anterior laxity. A common block to the reduction is represented by the interposition of the intermeniscal ligament (or jugal ligament) when present: in our experience, we never found the anterior horn of the lateral meniscus or the medial meniscus interposed, although described in the literature [9].

**Figure 1:**
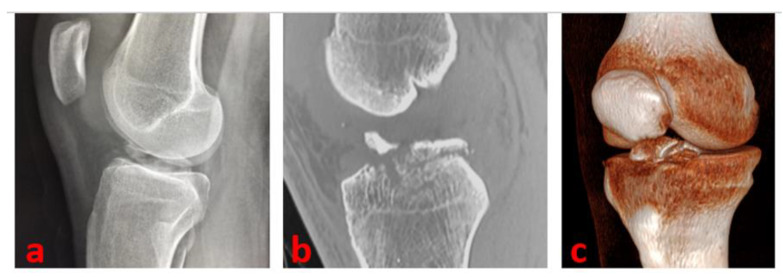
A true lateral knee radiographic view of the right knee of a 16-year-old male patient of our series shows a Type 3 tibial avulsion fracture (**a**); CT scans are useful to define the pattern of the fracture and drive the management: CT sagittal view scan (**b**) and 3D CT reconstruction (**c**) of the same patient.

A complete radiographic knee series with AP, lateral, tunnel, and Merchant views is a major support. A true lateral view with the knee in hyperextension is useful in determining the reducibility of the fragment; it helps us, especially in the case of type II avulsions. The radiographic series can be integrated with a CT scan (to further delineate the fracture pattern: size, shape, fragmentation, and comminution) and MRI (to assess for associated intraarticular injuries) [10]—(Figure 1).

Bony reduction in a displaced fracture has a relevant effect on knee stability since the tibial spine represents the ACL attachment site. For this reason, a displaced tibial spine is responsible for ACL deficiency. This important aspect should be considered in the treatment planning. The goal of the treatment of TSA is not only the restoration of congruity of the tibial plateau by obtaining an anatomic reduction but also the restoration of the ACL’s stabilizing role and the resolution of the mechanical block determined by the interposition of intraarticular soft tissues or by the fragments themselves [11].

First described by Poncet in 1875, this condition was largely illustrated by Meyers and McKeever in 1959, who outlined still-current classification and treatment guidelines [12]. Meyers and McKeevers’ classification can lead the decision process to define the more appropriate treatment (conservative or surgical option). A type I avulsion is a nondisplaced fracture. Type II avulsions are partially displaced fractures with an intact posterior hinge and a displacement of the anterior one-third to one-half of the eminence (also referred to as bird’s beak) [13]. In type III avulsions, the bony fragment is completely displaced. Type III avulsions are distinguished into two subtypes: IIIa fractures, with a complete separation of the fragment without apposition (involving the ACL insertion only), and IIIb, with a displaced and rotated cephalad fragment (involving the entire tibial eminence). Meyers and McKeevers’ classification was subsequently integrated by Zaricznyi in 1977, leading to type IV, describing an extremely comminuted intercondylar eminence [14]. While type I avulsions can be managed conservatively with knee aspiration (to reduce a tense haemarthrosis) and immobilization in a long-leg cast in a fully extended position [13]; undisplaced fractures need surgical management. The treatment of type II TSA is still controversial, and both conservative and operative options have been proposed [15]. Cast immobilization can be sufficient for patients whose fractures can be easily reduced through a closed reduction maneuver performed under radiological control [8,16]. Most of these fractures are not reducible due to soft tissue entrapment, and these situations drive surgical treatment [17]. Arthroscopy can play a diagnostic and sometimes even operative role [18].

In recent decades, different surgical options have been proposed to reduce and fix undisplaced TSA. Open reduction with internal fixation (ORIF) has found increasingly less space since arthroscopy is the preferred option to control and drive the reduction in the fragment and manage the associated injuries (meniscal or osteochondral lesions) [19]. Arthroscopic reduction and internal fixation (ARIF) can reduce soft tissue damage, wound complications, arthrofibrosis, post-operative pain, and the length of hospital stay compared to ORIF [1,20,21,22].

Concerning our experience, the TSA internal fixation approach began in 1991 with the use of metal staples as fixation devices, under arthroscopic control, based on the teachings of the undisputed pioneer Lanny L. Johnson. As widely reported in the literature, internal fixation can currently be performed by using metal or re-absorbable screws, sutures or suture anchors, bioabsorbable nails, double-loop endobutton devices, and stainless wires [23,24,25,26,27,28,29,30,31]. Fiber–wire suture fixation is the most popular arthroscopic fixation modality thanks to its ease of execution, despite less achieved stability if compared with other devices [32]. The choice is determined by the size and comminution of the bony fragment and the surgeon’s confidence, having three aspects in mind: the proximity to the proximal tibia growth plate, the preeminent role of the ACL in knee stability, and the potential limitation to knee motion if an optimal reduction is not obtained. A residual objective laxity (not corresponding to symptomatic knee instability) can be observed in many patients (maybe as the consequence of the elongation of ACL), even when anatomical reduction and stable fixation are performed [9,33].

Here, we describe the ARIF technique of TSA performed in our series (three patients from 30 December 2022 to 1 March 2023)—Type III TSA—with Kirschner wires through a mini-invasive approach in a simple, cheap, and reproducible modality. This technique allows a fast surgical and hospitalization time, a punctiform arthrotomy, and an early rehabilitation program and might favor the preservation of proximal tibial physis.

## 2. Materials and Methods—Surgical Technique

### 2.1. Ethical Statement

Written consent for the use of data for scientific purposes (including the anonymous use of pictures) was obtained from all patients as part of the standard pre-operative consent process of our institution, in compliance with the regulations in force concerning the ethical aspects. Data were collected as part of routine care, and permission has been given by our orthopedic institute. Internal Review Board approval was not necessary since case reports do not meet the legal definition of “research”—according to most interpretations; for this reason, ethical committee approval does not apply to this study.

### 2.2. Setup

**Figure 2:**
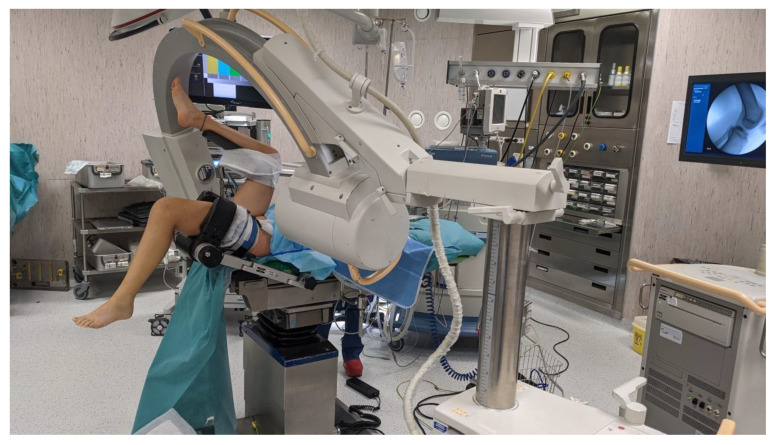
Operating theatre setup: the patient is in a supine position with the affected leg immobilized in a leg holder and the opposite leg held with a gynecologic leg holder to grant adequate workspace. C-arm fluoroscopy is set to radiographically guide fracture reduction and osteosynthesis with Kirschner wires.

The patient is placed in a supine position, and the surgical procedure is performed under locoregional anesthesia. The affected leg is immobilized in a circular leg holder with the knee flexed to 90°, and a tourniquet is applied at the root of the limb to improve anatomical visualization during arthroscopy. The opposite leg is held with a gynecologic leg holder to grant adequate workspace to the surgeons and adequate room for maneuver to the C-arm fluoroscopy, set in the operating theatre to radiographically visualize the fracture reduction and the positioning of the Kirschner wires (Figure 2).

### 2.3. Approach and Fracture Fixation

**Figure 3:**
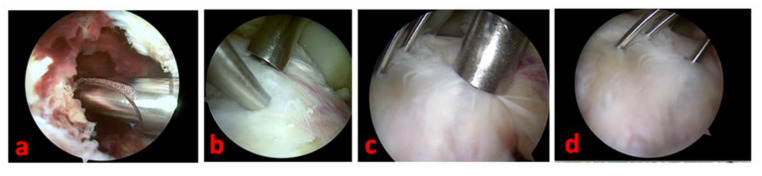
Arthroscopic view of tibial spine avulsion management of the left knee of a 16-year-old female patient—(**a**): light fracture site debridement is necessary to grant adequate visualization; (**b**): the reduction of the fracture is facilitated by using a less-invasive bone impactor; (**c**): a cannulated aimer is a useful tool to drive the Kirschner wires positioning; (**d**): the osteosynthesis is performed and completed by positioning three thin Kirschner wires in a cross-shaped geometry.

Standard anteromedial, anterolateral, and superolateral arthroscopic portals allow for full access to knee visualization, favoring a complete and direct visualization of the tibial eminence avulsion. We prefer to avoid the trans-tendinous Gillquist central portal [34], but we systematically use a modified Patel’s medial mid-patellar (superomedial) portal [35] or more patient-specific accessory ones if necessary to address associated intraarticular lesions. A thorough hematoma and blood clot washing and a selective Hoffa’s pat debridement are performed to achieve clear visualization of the joint. Care should be taken to avoid the complete removal of Hoffa’s pad to limit the post-operative knee stiffness. The eventual interposition of the intermeniscal ligament or the entrapment of the anterior horn of the medial or lateral meniscus in the fracture site can be easily managed by extracting them with an arthroscopic probe or a pick. The concomitant intraarticular lesions are arthroscopically addressed and managed according to the consolidated surgical modalities. We perform the debridement of the inflammatory tissue in the fracture site with a full-radius shaver blade and the reduction in the fracture by using a less-invasive bone impactor. With the knee flexed to 90°, the fracture fragments are then synthesized (under fluoroscopic control) with three thin Kirschner wires (maximum diameter: 2 mm) inserted in a proximal–distal direction in a cross-shaped geometry and guided by 2.4 mm cannulated aimers (Figure 3).

Cannulated aimers are also useful in performing a moderate compression of the fracture fragments while inserting the Kirschner wires. Two K wires are inserted through the superolateral portal to reduce the tibial intercondylar eminence (and mimic the orientation and the force lines of ACL fibers), and 1 K wire is inserted through the superomedial portal [35]. According to our experience, the Kirschner wires should ideally reach the opposite tibial cortical surface to strengthen the osteosynthesis mechanism. Caution must be taken to prevent iatrogenic damage to the external popliteal sciatic nerve; for this reason, we suggest pre-determining and equalizing the length of the Kirschner wires after the careful positioning of the first one under fluoroscopic control.

**Figure 4:**
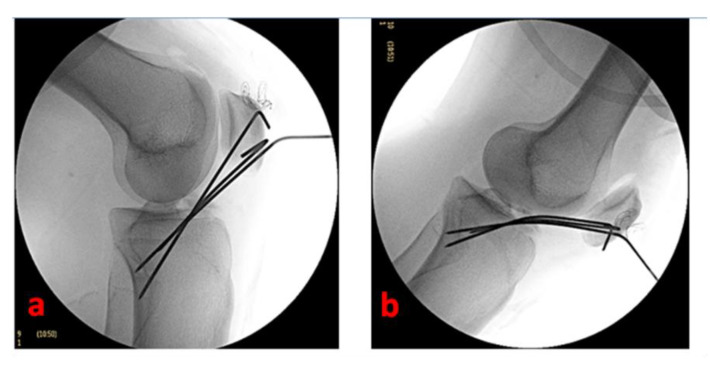
Fluoroscopic control of fracture reduction and osteosynthesis devices positioning of the left knee of a 16-year-old female patient with the knee flexed to 90° (**a**) and the knee completely extended (**b**).

Under arthroscopic and fluoroscopic visualization, we completely extend the knee to confirm the effectiveness of reduction and stability. The Kirschner wires are externally cut (by maintaining a 2–3 cm straight external portion—essential for the subsequent removal) and curved (Figure 4).

At this stage, the tourniquet is let down, the hemostasis performed, and the arthroscopic portals sutured. A compressive bandage and a long-leg cast at 30° of knee flexion (with the K wires anchored to the cast) are applied.

### 2.4. Post-Operative Management

Patients are instructed to maintain non-weight-bearing positions and to walk with two crutches for 3 weeks. Isometric quadriceps exercises are encouraged in the immediate post-operative phase. Clinical and radiographical controls can be performed 3 weeks post-operatively to evaluate and monitor the fracture healing process.

At this time, Kirschner wires can be easily removed with a drill in an outpatient setting under mild sedation using mini-invasive in-office needle arthroscopy (IONA) [18]. The timing of Kirschner removal privileges the need for the recovery of passive knee extension before complete fracture consolidation. Patients are then encouraged to progressively and gently increase weight-bearing and range of motion in the knee. A knee MRI is performed 40 days after the surgery; a complete renovation of the bone trabecular architecture (a sign of fracture restoration) has been seen in our experience.

**Figure 5:**
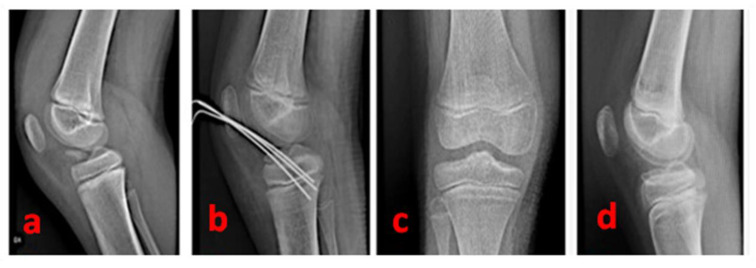
A radiographic summary overview of the right knee of a 9-year-old female patient—(**a**): pre-operative lateral view of a 3-type TSA; (**b**): post-operative radiographic control showing the cross-shaped geometry of the Kirschner wires; (**c**): antero-posterior radiographic control 15 months after surgery; (**d**): lateral radiographic control 15 months after surgery (complete restoration).

**Figure 6:**
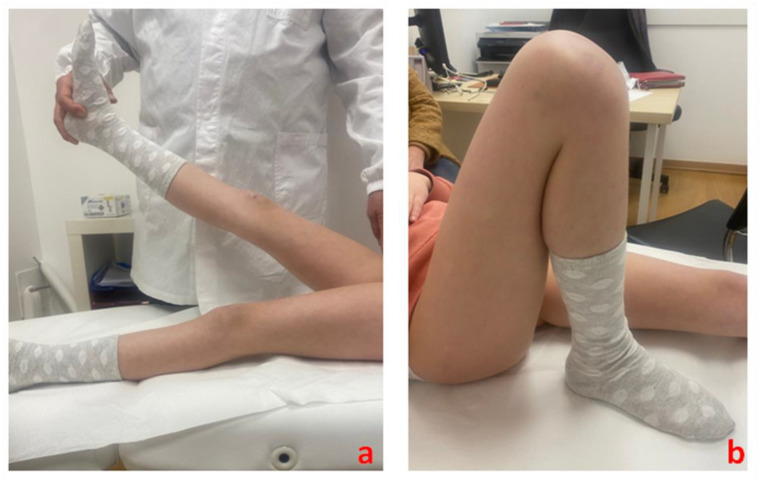
Three-month post-surgery clinical follow-up of the right knee of a 9-year-old female patient shows complete restoration of (**a**) full knee extension and (**b**) full knee flexion.

A complete clinical recovery (with a totally stable tibial spine) is generally obtained 3 months after the surgery. At this time, patients can be encouraged to resume sports activities (including skiing or cycling)—(Figure 5 and Figure 6).

## 3. Results

The technique proposed here has been used for the management of three patients of our series (a 9-year-old female, a 16-year-old male, and a 16-year-old female) within one week of the injury. Mechanisms of injury were sports-related; in two cases, the injury was caused by a fall while skiing, while in one case, injury occurred while cycling. All of the patients underwent preliminary clinical evaluation by the senior surgeon (V.C.) and underwent radiographic and MRI evaluation. According to the Meyers and McKeever classification, the fractures were classified as type III in all subjects. Among the three cases, the two female patients demonstrated Lachmann +++, Pivot shift ++, and anterior drawer ++, while the male patient presented as Lachmann ++, Pivot shift +, and anterior drawer +. All of the operations were performed by the senior surgeon with the arthroscopic technique described above, and the median operating time was 70 min (range, 60–80 min). All patients were discharged (with a long-leg cast immobilization with no weight-bearing) the day after surgery in good clinical condition. The radiographic and clinical controls performed 3 weeks post-operatively confirmed the satisfactory fracture healing process so that Kirschner wires could be easily removed with a drill in an outpatient setting under mild sedation. The follow-up physical examinations performed 3 months after the surgery demonstrated good clinical results in any of the patients, as confirmed by the highest scores on the Lysholm knee scale (100/100) and Oxford Knee Score (48/48), with no pain, no instability and complete functional recovery. The flexion of the injured knee was comparable to the healthy side in any of the patients; a 10° extension deficit was observed if compared to the contralateral knee (passive extension: 0° versus −10°) in only one patient (16-year-old female patient) without clinical limitations while it was the same as the healthy side in the other two cases. No complications, such as infection, algodystrophy, delayed fracture, or secondary fracture dislocation, were observed in our series during the follow-up time. The clinical outcomes at 3-month follow-ups were confirmed by the positive feedback of the radiographic controls. Considering the good clinical and radiographic results, all patients were encouraged to return to their common daily activities and to gradually resume their sport practice 3 months after surgery.

## 4. Discussion

Anatomical reduction and stable osteosynthesis of TSA are fundamental for successful fracture healing and restoration of knee stability and normal biomechanics. Different surgical techniques have been proposed in recent decades for the management of displaced tibial eminence fractures (Type II-III-IV according to Meyers and McKeever’s classification) [13,19,36,37]. Insufficient evidence is available to prefer or support one surgical technique over another [19]. The most common complications associated with TSA are residual laxity (often clinically asymptomatic), arthrofibrosis, fracture nonunion or malunion, osteosynthesis hardware prominence, and tibial physis damage (with growth arrest or deformity in pediatric patients). Arthrofibrosis (due to prolonged immobilization and/or longer surgical times) should be prevented by adopting an early aggressive rehabilitative protocol, both in conservative and post-surgical management. Fracture nonunion or malunion might be favored by an inadequate osteosynthesis technique (even determined by the interposition of the intermeniscal ligament or the anterior horn of the medial or lateral meniscus). Hardware prominence (common with screw osteosynthesis) can lead to extension deficit with notch impingement and may require implant removal when symptomatic. Growth disturbance in immature skeleton patients should be avoided or limited by using physis-sparing fixation techniques. Advances in diagnostic imaging, classification, and surgical techniques will likely reduce the occurrence of these complications in pediatric and adolescent patients [3,23]. Arthroscopic-based techniques have increased their popularity in recent decades due to their ability to minimize surgical morbidity, obtain a direct and clear visualization of the eminence avulsion, address intraarticular associated injuries, and obtain satisfactory clinical results. Furthermore, arthroscopy increases the ability to test ACL structural integrity (if compared to MRI performed in the acute phase), considering the remarkable mechanical stress to which ACL is subjected before the onset of the bony avulsion. Anyway, a gold-standard arthroscopy-based technique is still missing. In pre-operative planning, further attention must be put to preserving the tibial growth plate, given the higher frequency of these lesions in children and adolescents.

In this article, we describe a mini-invasive technique to reduce and stabilize displaced tibial eminence avulsions using Kirschner wires, suitable for skeletally immature patients.

This technique can be easily performed by surgeons routinary performing arthroscopic surgery. The combination of arthroscopic and fluoroscopic guidance allows you to achieve reduction and stabilization as accurately as possible. In our experience, this surgical technique, which requires a K wires fixation with the knee flexed to 90° and a temporary immobilization at 30° of flexion by bending the Kirschner wires, induces adequate compression of the fracture site and reparative osteogenesis stimulus. Furthermore, this osteosynthesis technique (unlike other methods) is only temporary so as to potentially avoid a potential leg length discrepancy caused by the eventual metaphyseal cartilage damage. Kirschner wires are generally used for the management of Salter–Harris pediatric fractures, having been considered minimally traumatic for the epiphyseal growth plate (though potential damage cannot be excluded in an absolute sense) [38]. On these premises, we supposed that these temporary and mini-invasive devices (with a maximum overall diameter of 6 mm) could respect the epiphyseal growth plates, being suitable for children and adolescents. Long-term follow-ups are needed to confirm our hypothesis. The reduced invasiveness of the osteosynthesis technique considerably limits the eventual risk of infection (as confirmed in our case series: no infection was observed).

Post-operative outcomes assessed in our experience show good clinical results at 3- and 6-month follow-ups. All patients obtained the highest scores on the Lysholm knee scale (100/100) and Oxford Knee Score (48/48) administered 3 months after surgery with no pain, a complete functional recovery, and the complete ability to perform common activities in daily life. In only one patient was a 10° extension deficit observed at a distance of time (>6 months) compared to the contralateral knee (passive extension: 0° versus −10°) without clinical limitations. An adequate rehabilitation program should start as soon as possible after cast removal to get a complete knee extension since we have observed an unavoidable tendency toward stiffness.

The limited cost of the implanted devices is a plus point that should not be neglected (in our experience, Kirschner wires, with a mean cost of 50–80 EUR/US Dollars, are considered a cheap solution, though unavoidable local differences related to price changes of single countries or institutions cannot be excluded). On the other side, the necessity of removing the fracture fixation devices could be considered a limitation of this procedure, though in our experience, it is an easy procedure executable in an outpatient context. Another limitation is represented by the expertise in arthroscopic surgery required to perform this technique.

We are aware of the evident statistical limit of our small series with short-term results, a limit shared with other studies reporting the outcomes of surgical techniques for the management of displaced TSA. A larger cohort of patients with a long-term follow-up (even examining the rate of risk of ACL future rupture) could help us to improve our knowledge of these fractures. Furthermore, a more detailed analysis concerning the overall cost-effectiveness of these techniques (compared to other methods) should be performed, but, in our opinion, it is beyond the scope of a technical note, requiring a different methodology.

## 5. Conclusions

Tibial eminence fractures can be treated with the described ARIF technique with Kirschner wires, which has been in our, experience, mini-invasive, safe, reliable, and cost-effective. The system we have here proposed seems attractive and very promising since it is respectful of the epiphyseal growth plates and is thus suitable for children and adolescents.

## Data Availability

All data generated or analyzed during this study are included in this article.

## Abbreviations

ACL	Anterior cruciate ligament
ARIF	Arthroscopic reduction and internal fixation
IONA	In-office needle arthroscopy
ORIF	Open reduction with internal fixation
TSA	Tibial spine avulsions

## References

[1] Coyle C., Jagernauth S., Ramachandran M. (2010). Tibial eminence fractures in the paediatric population: A systematic review. J. Child. Orthop. Surg..

[2] Wyatt J.D., Treme G., Veitch A.J., Johnson A.C. (2014). Incidence of Associated Knee Injury in Pediatric Tibial Eminence Fractures. J. Knee Surg..

[3] Salvato D., Green D.W., Accadbled F., Tuca M. (2023). Tibial spine fractures: State of the art. J. ISAKOS Jt. Disord. Orthop. Sport. Med..

[4] Patterson S.P., Christiansen G.B., Daffner R.H. (2018). Avulsion fracture of the tibial eminence in an adult with a unique mechanism of injury. Radiol. Case Rep..

[5] Yu D., Yu R., Zhang J., Chen T., Zhang B. (2020). Arthroscopic treatment of adult displaced tibial eminence fractures with anchor and pushlock fixation. Medicine.

[6] Rajanish R., Jaseel M., Murugan C., Kumaran C. (2018). Arthroscopic tibial spine fracture fixation: Novel techniques. J. Orthop..

[7] Noyes F.R., DeLucas J.L., Torvik P.J. (1974). Biomechanics of anterior cruciate ligament failure: An analysis of strain-rate sensitivity and mechanisms of failure in primates. J. Bone Jt. Surg..

[8] Scrimshire A., Gawad M., Davies R., George H. (2018). Management and outcomes of isolated paediatric tibial spine fractures. Injury.

[9] Kocher M.S., Micheli L.J., Gerbino P., Hresko M.T. (2003). Tibial Eminence Fractures in Children: Prevalence of Meniscal Entrapment. Am. J. Sport. Med..

[10] Green D., Tuca M., Luderowski E., Gausden E., Goodbody C., Konin G. (2019). A new, MRI-based classification system for tibial spine fractures changes clinical treatment recommendations when compared to Myers and Mckeever. Knee Surgery Sport. Traumatol. Arthrosc..

[11] Aderinto J., Walmsley P., Keating J. (2008). Fractures of the tibial spine: Epidemiology and outcome. Knee.

[12] Meyers M.H., McKeever F.M. (1959). Fracture of the Intercondylar Eminence of the Tibia. J. Bone Jt. Surg..

[13] Lubowitz J.H., Elson W.S., Guttmann D. (2005). Part II: Arthroscopic treatment of tibial plateau fractures: Intercondylar eminence avulsion fractures. Arthrosc. J. Arthrosc. Relat. Surg..

[14] Zaricznyj B. (1977). Avulsion fracture of the tibial eminence: Treatment by open reduction and pinning. J. Bone Jt. Surg..

[15] Adams A.J., O’hara N.N., Abzug J.M., Aoyama J.T., Ganley T.J., Carey J.L., Cruz A.I., Ellis H.B., Fabricant P.D., Green D.W. (2019). Pediatric Type II Tibial Spine Fractures: Addressing the Treatment Controversy With a Mixed-Effects Model. Orthop. J. Sport. Med..

[16] Shin Y.-W., Uppstrom T.J., Haskel J.D., Green D.W. (2015). The tibial eminence fracture in skeletally immature patients. Curr. Opin. Pediatr..

[17] Archibald-Seiffer N., Jacobs J., Zbojniewicz A., Shea K. (2015). Incarceration of the intermeniscal ligament in tibial eminence injury: A block to closed reduction identified using MRI. Skelet. Radiol..

[18] Arrigoni F., Mazzoleni M.G., Calvisi V., Masciocchi C. (2022). In-Office Needle Arthroscopy (IONA): May a traditionally orthopedic procedure enter the portfolio of interventional radiology?. Radiol. Medica.

[19] Osti L., Buda M., Soldati F., Del Buono A., Osti R., Maffulli N. (2016). Arthroscopic treatment of tibial eminence fracture: A systematic review of different fixation methods. Br. Med. Bull..

[20] Hamilton G.A., Doyle M.D., Castellucci-Garza F.M. (2018). Arthroscopic-Assisted Open Reduction Internal Fixation. Clin. Podiatr. Med. Surg..

[21] Yuan L., Shi R., Chen Z., Ding W., Tan H. (2022). The most economical arthroscopic suture fixation for tibial intercondylar eminence avulsion fracture without any implant. J. Orthop. Surg. Res..

[22] Tuca M., Bernal N., Luderowski E., Green D.W. (2019). Tibial spine avulsion fractures: Treatment update. Curr. Opin. Pediatr..

[23] Strauss E.J., Kaplan D.J., Weinberg M.E., Egol J., Jazrawi L.M. (2018). Arthroscopic Management of Tibial Spine Avulsion Fractures: Principles and Techniques. J. Am. Acad. Orthop. Surg..

[24] Gamboa J.T., Durrant B.A., Pathare N.P., Shin E.C., Chen J.L. (2017). Arthroscopic Reduction of Tibial Spine Avulsion: Suture Lever Reduction Technique. Arthrosc. Tech..

[25] Verdano M.A., Pellegrini A., Lunini E., Tonino P., Ceccarelli F. (2013). Arthroscopic Absorbable Suture Fixation for Tibial Spine Fractures. Arthrosc. Tech..

[26] Mutchamee S., Ganokroj P. (2020). Arthroscopic Transosseous Suture-bridge Fixation for Anterior Cruciate Ligament Tibial Avulsion Fractures. Arthrosc. Tech..

[27] Kobayashi S., Harato K., Udagawa K., Masumoto K., Jinnouchi M., Toyoda T., Niki Y. (2018). Arthroscopic Treatment of Tibial Eminence Avulsion Fracture with Suture Tensioning Technique. Arthrosc. Tech..

[28] Tang J., Zhao J. (2021). Arthroscopic Suture-to-Adjustable Loop Fixation of Adult Anterior Cruciate Ligament Tibial Avulsion Fracture. Arthrosc. Tech..

[29] Boutsiadis A., Karataglis D., Agathangelidis F., Ditsios K., Papadopoulos P. (2014). Arthroscopic 4-Point Suture Fixation of Anterior Cruciate Ligament Tibial Avulsion Fractures. Arthrosc. Tech..

[30] Sekiya H., Takatoku K., Kimura A., Kanaya Y., Fukushima T., Takeshita K. (2016). Arthroscopic Fixation with EndoButton for Tibial Eminence Fractures Visualised through a Proximal Superomedial Portal: A Surgical Technique. J. Orthop. Surg..

[31] Chu Y., Hu T., Chen M., Jiang C., Wu Z., Shi J. (2021). Preliminary clinical outcomes of the double-row anchor suture-bridge technique for the fixation of tibial intercondylar eminence fractures in adults: A 12-months minimal follow-up. BMC Musculoskelet. Disord..

[32] Dung T.T., Du H.G., Long N.H., Son L.M., Thanh D.X., Son D.N., Tuyen N.T., Van Minh D., Phương N.H., Nam V.T. (2019). Arthroscopic fixation of ACL avulsion fracture in the saint pault hospital: A review of treatment outcomes: Cohort study. Ann. Med. Surg..

[33] Willis R.B., Blokker C., Stoll T.M., Paterson D.C., Galpin R.D. (1993). Long-Term Follow-Up of Anterior Tibial Eminence Fractures. J. Pediatr. Orthop..

[34] Gillquist J., Hagberg G. (1976). A new modification of the technique of arthroscopy of the knee joint. Acta Chir. Scand..

[35] Calvisi V., Lupparelli S., Giuliani P. (2007). A View from Above: A Modified Patel’s Medial Midpatellar Portal for Anterior Cruciate Ligament Arthroscopic Surgery. Arthrosc. J. Arthrosc. Relat. Surg..

[36] Jackson T.J., Storey E.P., Ganley T.J. (2019). The surgical management of tibial spine fractures in children: A survey of the Pediatric Orthopaedic Society of North America (POSNA). J. Pediatr. Orthop..

[37] Callanan M., Allen J., Flutie B., Tepolt F., Miller P.E., Kramer D., Kocher M.S. (2019). Suture Versus Screw Fixation of Tibial Spine Fractures in Children and Adolescents: A Comparative Study. Orthop. J. Sport. Med..

[38] Slongo T. (2020). Technik und Biomechanik der Bohr-Draht(Kirschner-Draht)-Osteosynthese bei Kindern [Technique and biomechanics of Kirschner wire osteosynthesis in children]. Oper Orthop Traumatol..

